# Incidence of deformities and variation in shape of mentum and wing of *Chironomus columbiensis* (Diptera, Chironomidae) as tools to assess aquatic contamination

**DOI:** 10.1371/journal.pone.0210348

**Published:** 2019-01-10

**Authors:** Milton Leoncio Montaño-Campaz, Lucimar Gomes-Dias, Beatriz Edilma Toro Restrepo, Víctor Hugo García-Merchán

**Affiliations:** 1 Departamento de Ciencias Biológicas, Facultad de Ciencias Exactas y Naturales, Grupo de investigación BIONAT (Biodiversidad y Recursos Naturales), Universidad de Caldas, Manizales, Colombia; 2 Grupo de Investigación en Evolución, Ecología y Conservación (EECO), Programa de Biología, Universidad del Quindío, Armenia, Quindío, Colombia; Universidade Regional Integrada do Alto Uruguai e das Missoes, BRAZIL

## Abstract

Constantly, aquatic ecosystems are under pressure by complex mixtures of contaminants whose effects are not always easy to evaluate. Due to this, organisms are sought in which early warning signs may be detected upon the presence of potentially toxic xenobiotic substances. Thereby, the study evaluated the incidence of deformities and other morphometric variations in the mentum and wing of *Chironomus columbiensis* exposed to water from some of the Colombian Andes affected by mining, agriculture, and cattle raising. Populations of *C*. *columbiensis* were subjected throughout their life cycle (24 days) for two generations (F_1_ and F_2_). Five treatments were carried out in controlled laboratory conditions (water from the site without impact, site of mining mercury, mining mercury + cyanide, cattle raising, and agriculture) and the respective control (reconstituted water). Thereafter, the percentage of deformities in the mentum was calculated, and for the morphometric analysis 29 landmarks were digitized for the mentum and 12 for the wing. As a result, four types of deformities were registered in the *C*. *columbiensis* mentum, like absence of teeth, increased number of teeth, fusion and space between teeth, none of them detected in the individuals from the control. Additionally, the highest incidence of deformity in F_1_ occurred in the treatment of mining mercury, while for F_2_ this took place in the treatments of mining mercury + cyanide, cattle raising and agriculture. Differences were also found with respect to the morphometric variations of the mentum and wing of *C*. *columbiensis* among the control and the treatments with water from the creeks intervened. The treatments of mining mercury + cyanide and agriculture had the highest morphological variation in the mentum and wing of *C*. *columbiensis*. The results suggest that the anthropogenic impacts evaluated generate alterations in the oral apparatus of the larval state of *C*. *columbiensis* and in the adult state provoke alterations in the wing shape (increased width and reduced basal area). These deformities may be related to multiple stress factors, among them the xenobiotics metabolized by the organisms under conditions of environmental contamination.

## 1. Introduction

Increased human population and its expansion in rural areas has generated increased production and deposit of dangerous wastes into aquatic ecosystems as a result of intensified industrial, mining, agricultural, and cattle raising activities [1]. Diverse authors have revealed that the different soil uses can affect fauna and flora at molecular, cellular, morphological, physiological levels [2, 3]. In fact, literature reports that the presence of toxic substances in hydric resources generates an increase of structural deformities in the members of the aquatic communities, such as insects, gastropods and fish [4, 5, 6], affecting in the long term the dynamics of these ecosystems [7].

Different authors indicate that morphological variations in aquatic individuals can be an indication of contamination and, thereby, can be considered a potential tool to evaluate the quality of water sources [8, 9, 2, 6]. Within this context, it is important to have a precise method in the comparative analyses of the morphology of the organisms and their possible variations, as well as the geometric morphometrics that becomes a precise tool when quantifying the morphological variation of a structure [10, 11, 12, 13]. This method permits analyzing the variations in the shape of the organisms through a configuration elaborated based on homologous points (landmark) of the taxa [14], which are abstracted in geographic coordinates, which permits comparing details in the shape of the structures of the population studied. Hence, one of the big advantages of this tool is that it permits studying independently variations in the shape and size, guaranteeing a coherent interpretation of the results [15]. Additionally, through symmetry tests small deviations can be measured that occur between the left and right sides of bilateral symmetrical traits of the organisms that can be caused by conditions of environmental stress [16, 17, 18, 19, 20, 21].

Among the taxa broadly distributed in freshwater ecosystems, there are chironomidae and different studies have revealed the high degree of sensitivity of *Chironomus* to the presence of xenobiotics, which alter some of their biological traits, especially phenotypic traits [22, 23, 24, 25, 26, 27, 6]. Among the morphological parts affected most by toxic agents, there are antennae, mandibles, pre-mandibles, labrum laminae, wings, and mentum [28], with the last two being the most used in toxicological studies [29, 2, 30, 27, 6, 28]. The mentum of *Chironomus* is a structure located on the ventral part of the cephalic capsule with 13 sclerotized teeth distributed into six lateral teeth on both sides of the medial trifid tooth (Warwick & Tisdale, 1988). This structure in presence of contamination can present deformities, like addition, division, lack, notches, wear, and deviation of the teeth [22, 23, 31, 25, 26, 2, 30, 27, 6]. According to studies, the shape of the wings of insects can act as an early warning of stress in the development of the insects [32], given that these require many genes acting throughout their development [33] reflecting big changes in wing shape during their development even with slight disturbances [34].

Considering the morphological response of organisms to toxic agents and that the region of the Colombian central Andes is a highly populated zone and intervened by anthropic actions, like gold mining, cattle raising, and coffee and vegetable crops, this study sought to evaluate the incidence of deformities and the morphological variation of the mentum and wings of *C*. *columbiensis* in some zones of the Colombian Andes affected by the activities mentioned.

## 2. Materials and methods

### 2.1 Study area

The hydrographic basin of the Chinchiná River is located in the south-central region of the department of Caldas, on the western slope of the Central mountain range. It extends between 05°07’05.3” North and 75°40’10.3” West, at 800 m at its river mouth in the Cauca River, and 05°03’30” North and 75°23’03” West, at 5200 m in Laguna Negra, inside Los Nevados Natural National Park [35]. The zone covers approximately 113,250 ha, where cattle raising, agriculture, and mining are among the principal anthropic activities. Its tropical climate is characterized by small fluctuations of the inter-annual temperature but has considerable daily fluctuations and a bimodal rain distribution during the year. Mean annual precipitation is between 1800 and 2200 mm/year with maximum during April-May and August-November; its temperature fluctuates between -3°C and 29.2°C, determined by altitude [35].

This work considered five sampling sites, according to land use. One site without visible anthropic impact (1), two with gold mining (2 and 3), one with cattle raising activity (4), and another with agricultural activity (5). Site E1 (05°03'10.9''N, 75°24'33.6''W) was a zone without anthropic impact in La Elvira creek, located up water from an area of mining exploitation. It has transparent water and riparian vegetation where shrub and arboreal species predominate. Site E2 (05°03'4.4''N, 75°24'33.1''W) is a zone with mining-mercury activity located down water from E1 and has visible effects due to gold mining activities. It has grey murky waters and the riparian vegetation includes pastures and some terrestrial herbs. Site E3 (5°1'53''N; 75°24'43.8''W) is in the Manizales creek, has mining mercury-cyanide activity and is located down water from E2. It has visible effects due to gold mining activities and extraction of river material, with dark grey murky waters, with prevalence with prevalence of typical vegetation from pastures, like grass, terrestrial herbs, and some shrubs. Site E4 (05°04'32"N, 75°24'60"W) with cattle raising activity is located in the Cimitarra creek. This place has transparent waters and prevalence of pastures. Site E5 (05°01'50.79''N, 75°31'39.59''W) with agricultural activity is located in the Don Alonso creek (Villamaría). It has dark brown water and is immersed in a matrix of vegetable crops, with riparian vegetation where pastures and herbaceous predominate. Specimen collection permits were regulated by resolution 1166 of October 9, 2014 issued by the National Environmental Licenses Authority (ANLA) of Colombia and by decree 1376 of June 27, 2013 from Ministry of Environment and Sustainable Development of Colombia.

### 2.2 Test organism and culture conditions

Samples of *Chironomus columbiensis* (the species is not under any category threat according to the IUCN Red List of Species) were obtained from cultures prepared since 2012 in the Zoology laboratory in Universidad de Caldas. The culture was kept isolated with a photoperiod of 12 h of light 12 h of darkness at 23 ± 2°C, and the bottom had crushed disposable towels as substrate. The aquariums were filled with semi-soft reconstituted water (S1 Table), which had a pH = 7.2 ± 0.3, conductivity = 177 μS/cm^2^, and hardness = 138 mg CaCO_3_/l. Masses of freshly laid eggs were removed from the cultures and were placed to hatch in an aquarium with the same conditions as the culture, but without substrate. After the eggs hatched, individuals were obtained from the first larval instar, which lasted approximately 96 h.

### 2.3 Water sampling and physical, chemical, and hydrological variables

Three samplings were conducted during February, July, and November 2014, measuring *in situ* the physical-chemical variables of water temperature (°C), pH, conductivity (μS), and dissolved oxygen (mg/L). The water collected for the tests was stored in 10-L containers and kept refrigerated until the start of the tests. Water was also sampled from each creek for later *ex situ* determination of the oxygen chemical demand (OCD), oxygen biological demand (OBD5), total coliforms, fecal coliforms, total solids (TS), total suspended solids (TSS), cyanide (CN), boron (B), lead (Pb), mercury (Hg), ammonia nitrogen (NH3-N), phosphates (PO4), sulfates (SO4), iron (Fe), chlorides (Cl-), fats and oils, nitrates (NO3), nitrites (NO2), and aluminum (Al) (Annex 1). All the samples were taken by following protocols for sampling and preservation of water physical-chemical samples (properly refrigerated). The hydrological variables of depth (cm), width (m), and flow rate (m/s) were taken *in situ*, according to that proposed by Chará (2003) [36] for biological evaluation of aquatic environments. Furthermore, during the experiment, oxygen, conductivity, pH, and temperature were monitored at the beginning and in four-day intervals until the end of the experiments.

### 2.4 Experimental aquariums and sample preparation

Laboratory standardized conditions were maintained. Each aquarium was added two sheets of crushed disposable towels as substrate and 5 L of water from the control and from the study sites, with soft aeration. The water and substrate were left to interact for two days to permit toxic equilibrium and sedimentation of solids in suspension according recommendations by the OCDE guidelines for bioassays [37].

The study design included two aquariums for each treatment repeating the experiment three times. Four days before the experiments, six masses of freshly laid eggs were removed from different laboratory cultures and were placed in a small aquarium with 2 L of breeding water, bearing in mind that spawning takes more or less three days to hatch and, thus, guarantee that samples were in their first larval instar (less than 72 h since birth). Each treatment selected randomly 60 larvae, and these were fed with 0.3 g TetraMin twice per week. The experiments were prolonged until oviposition and death of the adults, thereby, obtaining larvae from the second generation of less than 72 h since birth.

### 2.5 Assembly of the mentum and wing

Fifteen samples from the fourth larval instar were removed from each aquarium and deposited onto a Petri dish with alcohol at 10% to avoid damage in the structures. After 10 min, the alcohol was changed to 96% to conserve the samples in perfect condition. Ninety larvae per generation and 180 larvae per treatment and from the control, for a total of 1080 larvae dissected, had their cephalic capsules removed. According to the Pinder protocol (1983) [38], from each treatment and generation assemblies were made of the cephalic capsules in ventral view on slides using Euparal. The cephalic capsules were photographed and analyzed in a Nikon Eclipse LV100 D-U microscope and a NikonSigh DS-FI1-U2 digital camera.

For the wings, 180 samples were selected (90 for each generation, 45 males and 45 females). The left wing was extracted from 1080 adult samples; this was extended on a slide with alcohol; thereafter, the coverslip was placed, and sides were sealed with transparent tape. The wings were photographed in a Nikon SMZ 1500 stereomicroscope and a NikonSigh DS-FI1-U2 digital camera.

### 2.6 Incidence of deformities in the mentum

**Fig 1:**
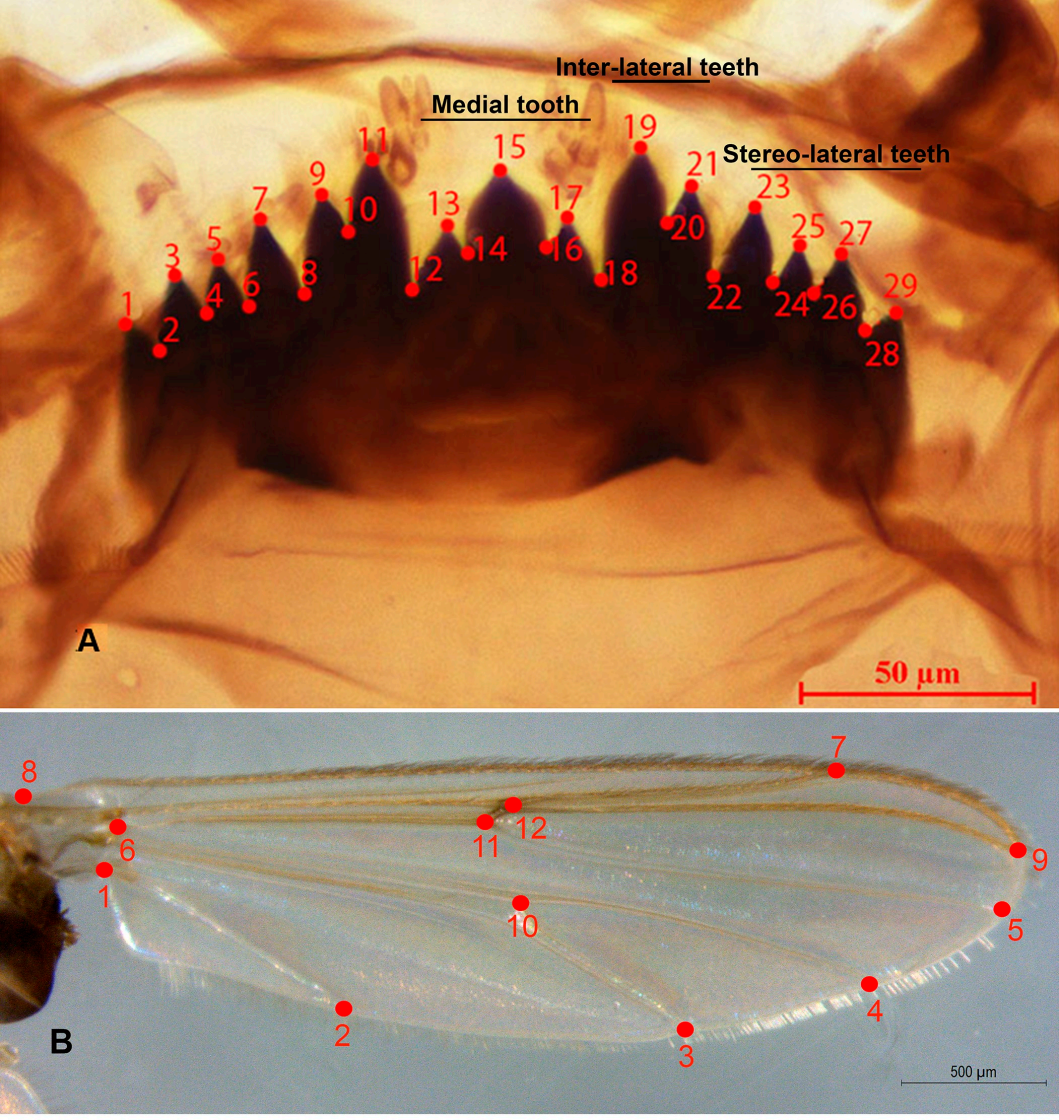
Digitized reference landmarks for morphometric analysis of *Chironomus columbiensis*. A) mentum; B) wing.

To evaluate the incidence of deformities in the samples from each treatment, initially the type of deformity was established by following Salmelin *et al*., (2015) [27] and considering only absolute deformities, that is, starting from a normal mentum (Fig 1A). It was considered deformed if it had additional, lacking, or fused teeth or Köhn’s gap, that is, a space between the teeth (wear was not considered deformity). To compare the rate of deformity among the different treatments and the control and among generations, a test was performed for the differences of proportions through the InfoStat statistical software version 2008.

### 2.7 Morphometric analysis of the mentum and wing

To quantify the possible differences in the shape and size of the mentum of the individuals, 29 landmarks or type-1 points of reference were digitized (Fig 1A), according to the methodology by Bookstein [11] and Arambourou [2, 30]. To analyze the wing, 12 landmarks were kept in mind (Fig 1B), according to Hoffmann *et al*. [29] & Arambourou [2]. This stage considered the criteria of positional homology, consistency in the relative position, adequate coverage of the shape, and repeatability [13], using the tpsUtil and tpsDig2 software [39]. Upon digitalization the landmarks, the data were registered in configuration matrices, [40]. After obtaining the matrix, the translation, scale, and rotation effects were eliminated by using the Procrustes superimposition generalized method [10]. Thus, information was obtained from a reference configuration of the average shape from the populations evaluated to be used in later analyses [41, 42]. As exploratory method, a covariance matrix was constructed and from such principal component analyses (PCA) were performed, which permitted constructing the morpho-space and identifying the position of the individuals from different treatments in function of the shape of the mentum and wing.

To evaluate the intra-observer measurement error, 30% of the structures of the samples evaluated were digitized, using the same configurations on two occasions for the mentum and wing. With these coordinates, a Procrustes ANOVA [17, 43] was performed with the MorphoJ software [44] to obtain the percentage of measurement error of the size and shape of the mentum and wing.

To evaluate the variation of the mentum and wing shape, two configuration matrices were constructed aligned with the landmarks (= LMs); one with the left side of the mentum and another with the left side of the wing. Analysis of data normality was performed, which presented normal distribution. Thereafter, ANOVA was performed to measure the effect of each treatment on the variation in shape and size independently for the mentum and for the wing [43, 45]. Thus, the statistical significance was estimated of the effects per treatments and generation. To test the effects of size on the shape variation, regressions were carried out according to Klingenberg & Marugán-Lobón [46], taking the differences obtained from the coordinates of the adjusted Procrustes of the mentum and wing (shape variable) as the dependent variables and the difference in the logarithm of the size of the centroid (size variable) as the independent variable.

The model was evaluated through a statistical test of permutations with 10,000 randomizations, which assumed the null hypothesis of independence of the shape with respect to size. To visualize the appearance of deformities of the shape of the mentum and wing, a CVA was performed in the MorphoJ program [4, 47, 45]. Finally, to establish if significant differences existed among treatments and controls and identify which treatment had differences compared with the control, Dunnett’s test was applied, with the CVA values in the SPSS PASW program version 18.

To estimate the asymmetric variation of the shape of the mentum, configuration matrices were used aligned through the Procrustes superimposition (= LMs) from the left side and right side and an ANOVA Procrustes was executed [43, 45]. Thus, the statistical significance was estimated from the effects of the fluctuating asymmetry, occurrence of directional asymmetry, or anti-asymmetry by treatments and per generation. Following the proposal by Klingenberg *et al*., [48], this analysis was accompanied by the calculation of the magnitude of the asymmetry of each measurement, like the percentage of the difference resulting from the ratio between the average difference of the left-side measurements minus the right-side measurements over the average value from the left side (Average(left-right)/(Average(left))*100.

Nevertheless, the assumption of isotropic variation is not very realistic in most biological data, which is why the procedure was complemented with a multivariate analysis of variance (MANOVA) that permit avoiding said assumption [49]. The MANOVA was conducted by following an F distribution and calculating its respective *P*. To test the effects of size in the asymmetric variation of the shape, regression analyses were performed according to Klingenberg & Marugán-Lobón [46]. To visualize the asymmetric variations from the left side and right side of the mentum, a CVA was carried out obtained from the covariance matrix of the effect of the sum of squares, in the MorphoJ program [43, 47, 45]. Finally, to establish if significant differences existed between treatments and controls and identify what treatment is presenting differences compared with the control, Dunnett’s test was conducted with the values of the CVA with the SPSS PASW program version 18.

### 2.8 Sampling sites and their relation with environmental variables

To determine the relation between the physical-chemical and hydrological variables and the treatments evaluated, a PCA was performed with the RStudio software (Version 0.99.484). The work kept in mind hydrological variables (O_2_, flow, pH, and conductivity) and physical-chemical variables (OCD, TS, NO_2_, GyA, and Cl), which were elected according to their significance through the Spearman correlation test, which removed those environmental variables with a high correlation (>0.8 and p ≤0.05). Furthermore, the altitude of each of the creeks was considered (sampling sites).

## 3 Results

### 3.1 Deformities of the *C*. *columbiensis* mentum

**Fig 2:**
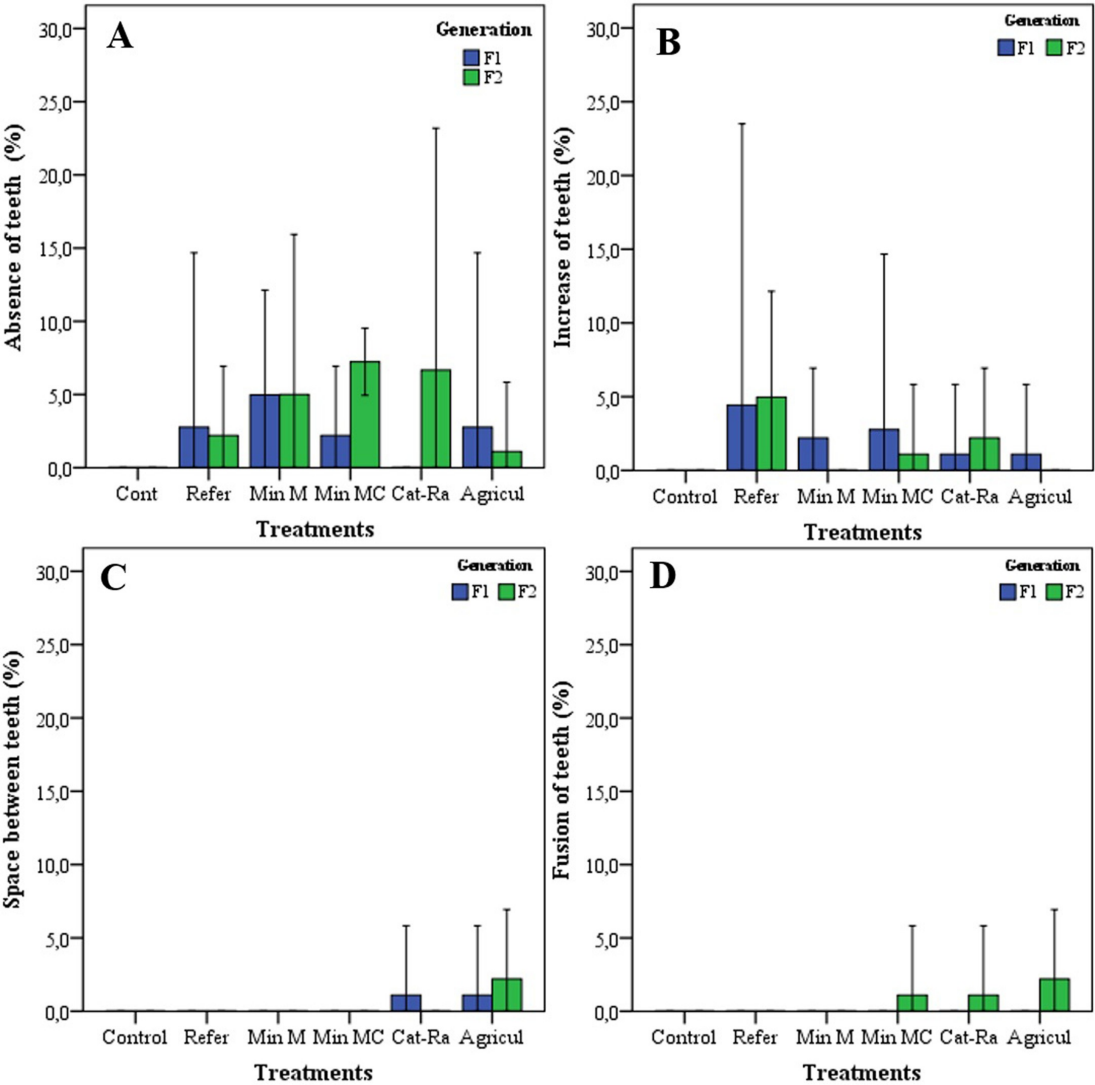
Deformities of the *Chironomus columbiensis* mentum per treatments and generation. A) absence of teeth; B) increase of teeth; C) space between teeth; D) fusion of teeth.

The study registered four types of deformities in the mentum of *C*. *columbiensis* only in the treatments; the control did not show any deformities. The deformities found were absence of teeth, increased number of teeth, fusion and space between teeth (S1–S4 Figs). The most frequent type in all the treatments was the absence of teeth and increased number of teeth, space between teeth and fusion of teeth were the least-frequent deformities (Fig 2).

Significant differences were found in mentum deformities in F_1_ between the control and the treatment with water from the site affected by mining mercury (differences of proportions test; *p* = 0.03). In contrast, no differences were found in mentum deformities in F_1_ among the other treatments and the control (differences of proportions test; *p* >0.05). In F_2_ significant differences were noted among the treatments with water affected by mining mercury-cyanide, cattle raising, and agriculture with the control (differences of proportions test; *p* <0.05). In tests with samples of water affected by mining mercury and the reference site, no differences were observed with the control (differences of proportions test; *p* >0.05). In addition, the greatest deformity in F_1_ was registered in the treatment with water from the site with mining mercury (6.9%) and in F_2_ in the assay with water from the site with cattle raising with 12.5% (Table 1).

**Table 1:** Rate of deformity (%) of the *Chironomus columbiensis* mentum by treatments and generation. N = number of samples per treatment and per generation; M = mercury; MC = mercury-cyanide.

Generation	Treatment					
	Control	WAI	Mining M	Mining MC	Cattle raising	Agriculture
F1	0.00	5.56	6.94	4.17	2.78	4.17
F2	0.00	4.17	4.17	9.70	12.50	6.94
N = 72						

### 3.2 Morphometric variation of the mentum and wing

Landmark digitalization error was at 0.4% for size and 0.7% for shape of the mentum and wing of *C*. *columbiensis*, which are acceptable values, given that they do not exceed 10%. The analysis of variance revealed significant differences in the shape of the *C*. *columbiensis* mentum among the different treatments (*p* <0.000), but not among generations (*p* >0.05). The control group was different from all the treatments (*p* = 0.000), similar results were found for the reference site (*p* <0.01). The permutation test conducted on the regression between the centroid size and the shape vectors did not indicate any allometric effect on the fluctuating asymmetry in the *C*. *columbiensis* mentum (*p* >0.05), where size has a variation below 10%.

**Fig 3:**
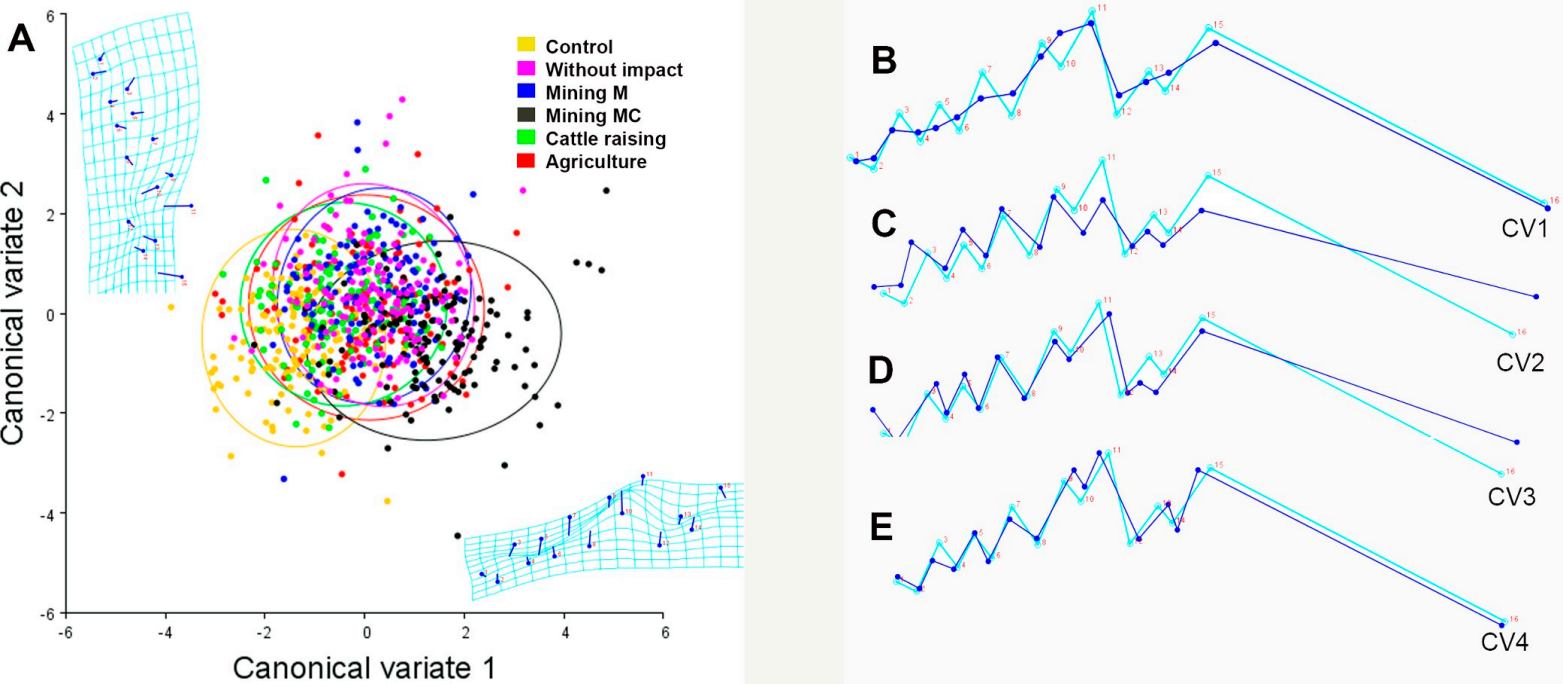
Canonical variate analysis in the shape of the *Chironomus columbiensis* mentum. A) Explain 82% of the total variance of the data in its two first exes, ellipses = 95%CI. B–E shows the shape variation of the mentum of *Chironomus columbiensis* in its four first canonic axes, which explain 95.5% of the total variance of the data. Light blue line: consensus composition of the shape. Dark blue line: disparities of the shape represented in each component. CV1: 68.2%, CV2: 13.8%, CV3: 7, and CV4: 6.5%. M = mercury and MC = mercury-cyanide.

The CVA of the mentum shape explained 82% of the variance with the first two canonical axes (Fig 3A). The positive end of the CV1 grouped the individuals treated with water from the site affected by mining mercury-cyanide; those from the control group were grouped toward the negative CV1 axis. Results of the samples from the assays with water affected by mining mercury, cattle raising, agriculture, and of reference are grouped in the neutral point of CV1 and CV2.

Fig 3B–3E shows the shape variation of the mentum, represented in the four first canonic axes that explain 95.5% of the total variance. The CV1 with blunt teeth that lose sharpness (Fig 3B) represents the principal pattern of the mentum shape variation, with 68.2% of the total data variance. This pattern of the morphological variations occurs in all the treatments, separating them from the control group. The CV2, with 13.8% of the total mentum shape variation, consists in increased size of the lateral teeth and loss of sharpness of the inter-lateral teeth and of the central trifid tooth (Fig 3C). This pattern of morphological variations occurs in samples from systems affected by mining mercury, cattle raising, agriculture, and the site without anthropogenic impact (WAI), separating them from the control group. The CV3, with 7% of the total mentum shape variation, is altered by an increase in the length of the first two lateral teeth (Fig 3D). This pattern differentiates the samples treated with water from the site of cattle raising and that from the control. Lastly, CV4 –with 6.5% of the total mentum shape variation–consisted in an increase of the length and reduction of the width of the inter-lateral teeth (Fig 3E) and differentiates the agricultural treatment from the control group.

The ANOVA and MANOVA revealed significant differences in the fluctuating asymmetry of the *C*. *columbiensis* mentum among the different treatments and control (*p* <0.0001), but not among generations (*p* >0.6737). Furthermore, directional asymmetry was detected in all the treatments (*p* <0.0001); teeth on the left side of the mentum were frequently less sharp than those on the right side. The control group was different from all the treatments (*p* = 0.000). Similar results were found for the reference site (Dunnett’s test; *p* <0.001), with greater variation in individuals from the treatment with water from the sites with mining mercury-cyanide, agriculture, cattle raising, mining mercury, and reference site, in their order. The permutation test conducted on the regression between the centroid size and the shape asymmetry vectors did not indicate any allometric effect on the fluctuating asymmetry in the *C*. *columbiensis* mentum (*p* >0.05), where size had a variation below 10%.

**Fig 4:**
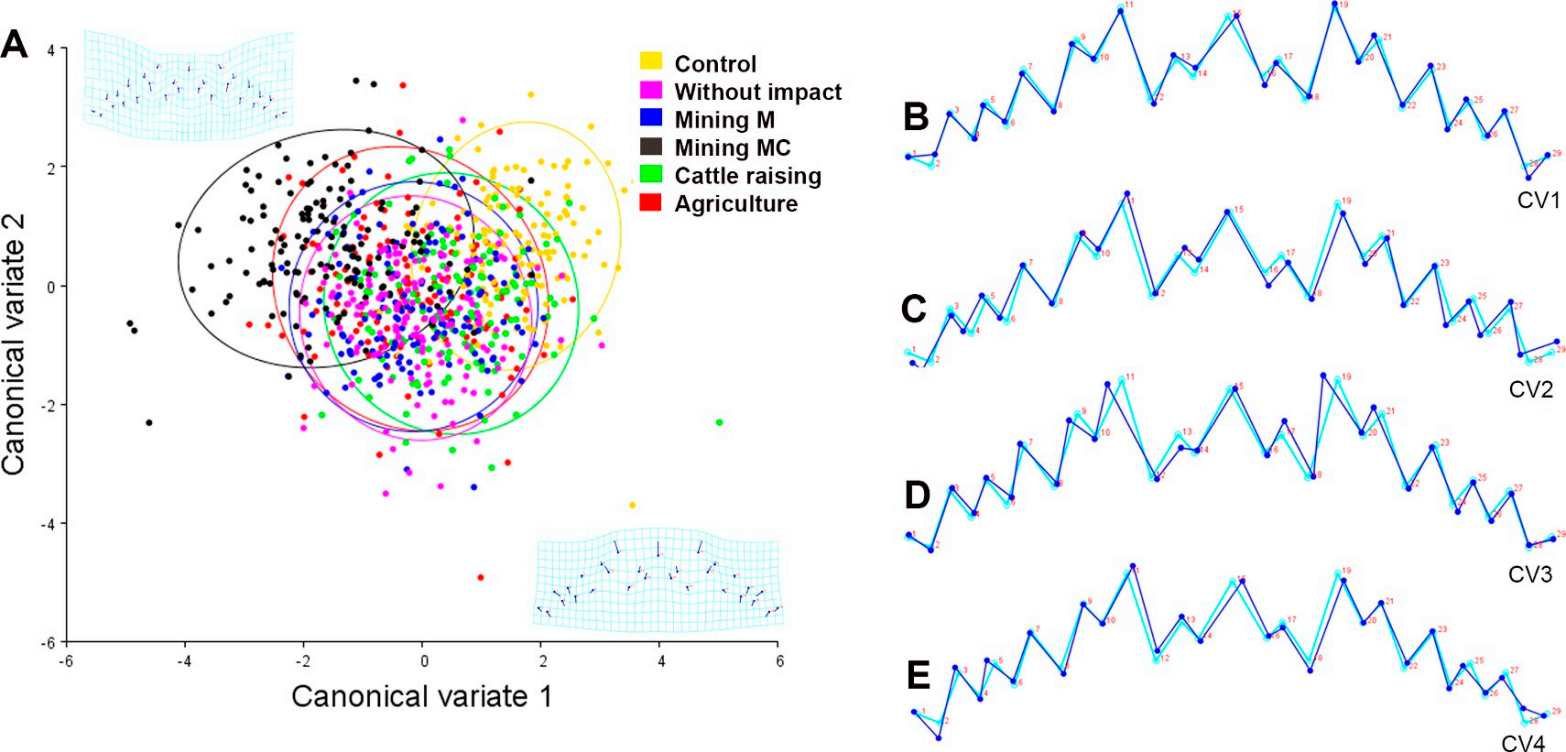
Canonical variate analysis of the fluctuating asymmetry of the *Chironomus columbiensis* mentum. A) Explain 83.04% of the variance of the data in its two first exes, ellipses = 95%CI. B–E shows the fluctuating asymmetry of *Chironomus columbiensis* in its four first canonic axes, which explain 93.4% of the total data variance. Light blue line: consensus decomposition of the shape. Dark blue line: disparities of the shape represented in each component. CV1: 39.8%, CV2: 21.3%, CV3: 17.7%, and CV4: 14.6%. M = mercury and MC = mercury-cyanide.

The canonical variate analysis of the fluctuating asymmetry explained 83% of the variance with the first two canonic axes (Fig 4A). The positive end of CV1 grouped individuals from the control, while individuals from the group of mining mercury-cyanide were grouped towards the CV1 negative axis. Samples from the treatments with mining mercury, cattle raising, agriculture, and of reference are grouped in the neutral point of the CV1 and CV2.

Fig 4B–4E shows the variation of the mentum’s symmetrical shape, represented in the first four canonic axes that explain 93.4% of the total variance.

The CV1, with 39.8% of the total data variance, illustrates that the principal deformity consists in a reduction of the first left lateral tooth and an increase of the last right lateral tooth. Additionally, a slight increase is observed of the left lateral trifid tooth, along with a slight reduction of the right (Fig 4B), principally in treatments with mining mercury, mining mercury-cyanide, agriculture, and the reference site, presenting significant differences with the control group. The CV2, with 21.3% of the total variance of the mentum shape shows, mainly in the agriculture treatment, displacement of the trifid tooth and the inter-lateral teeth to the left side of the mentum with greater proportion from the right side (Fig 4C). The CV3, with 17.7% of the total variance, reflects an increase of the left trifid tooth and a reduction of the right (Fig 4D) in treatments with mining mercury, mining mercury-cyanide, and agriculture. Lastly, CV4, with 14.6% of the total data variation, illustrates an increase of the lateral teeth from the left side and a reduction of the right (Fig 4E) in treatments with mining mercury and cattle raising.

**Fig 5:**
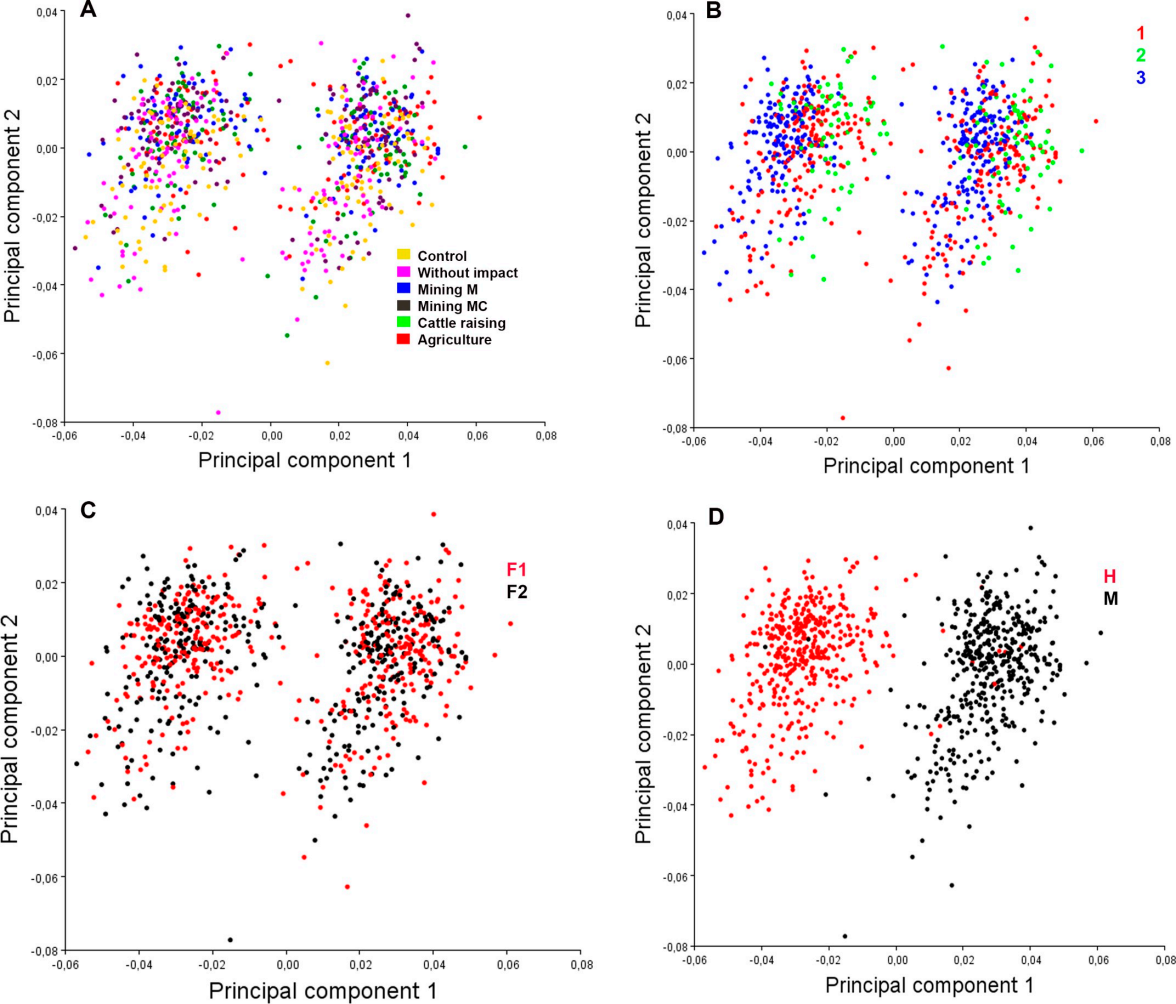
Principal components analysis of the wing of *Chironomus columbiensis* showing the sexual dimorphism among the individuals. M = mercury and MC = mercury-cyanide. Ellipses = 95%CI. A) Individuals per treatments; B) Individuals per experiments; C) Individuals per generation; D) Individuals per gender.

**Fig 6:**
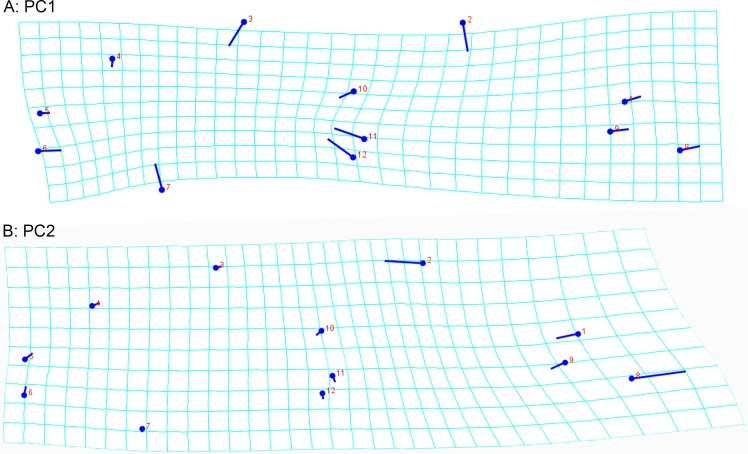
Deformations of the wing shape of males and females of *Chironomus columbiensis* represented in the first two (A,B) principal components.

With respect to the wing’s shape variation, analyses of principal components suggest the existence of sexual dimorphism in the wing of *C*. *columbiensis* adult individuals (Fig 5). In addition, sexual dimorphism does not alter the results of the treatments (Fig 5A), experiments (Fig 5B), and generations (Fig 5C) when working with equal numbers of males and females (Fig 5D). Fig 6 shows the points that represent the shape consensus and the deformation vectors, showing the magnitude and direction of the partial deformations represented in the first two principal axes. The PC1, with 66.6% of the total variance, indicates that the principal deformity consists in the wing’s width reduction in males, while the PC2 (16.4%) shows a reduction of the wing’s basal part in females (Fig 6).

**Fig 7:**
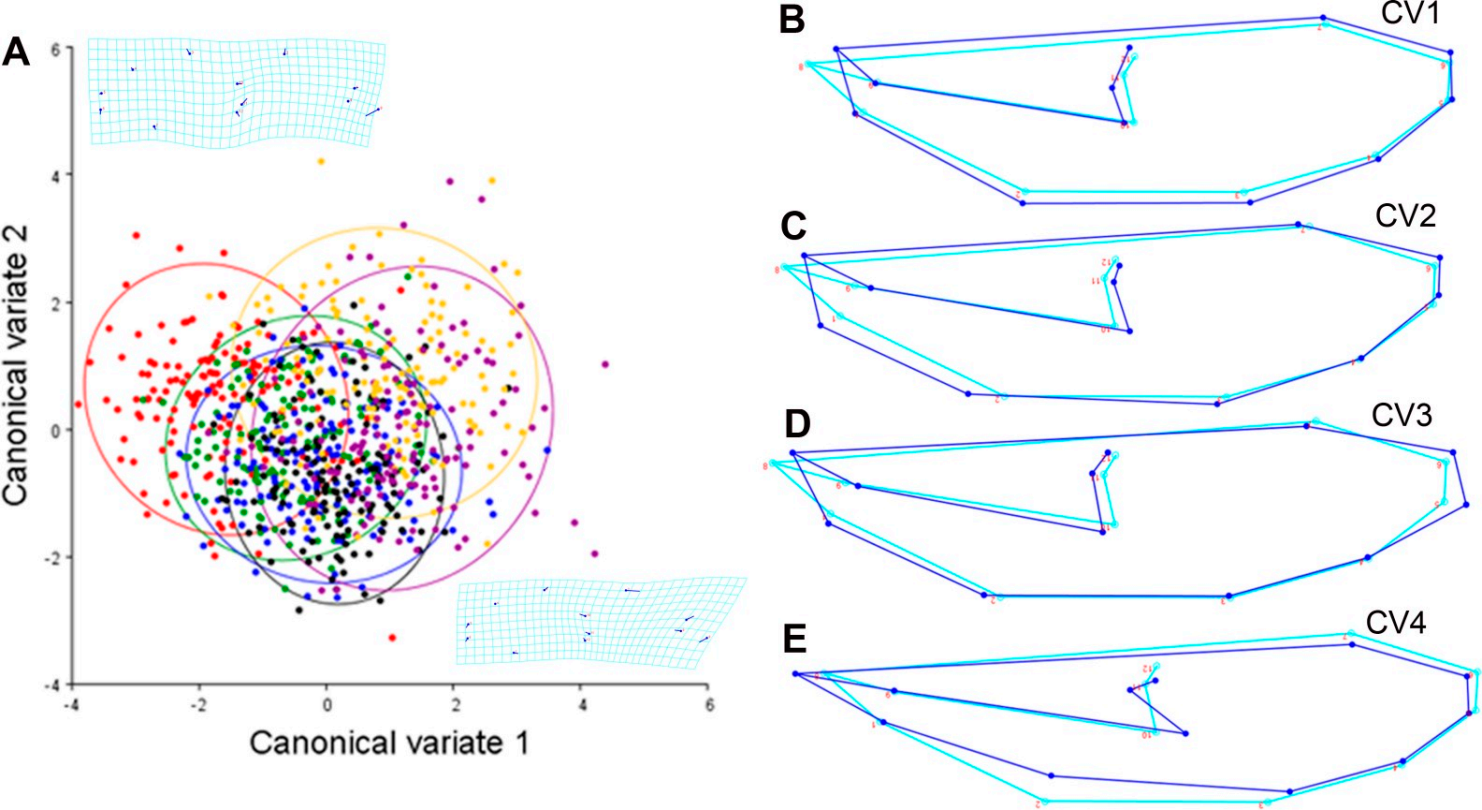
Canonical variate analysis of the *Chironomus columbiensis* wing. A) Explaining 90.3% of the variance of the data in its two first exes, ellipses = 95%CI. B–E shows the deformations represented in the first four canonic analyses, explaining 98.7% of the variance. Light blue line: consensus decomposition of the shape. Dark blue line: disparities of the shape represented in each component. CV1 = 60%, CV2: 22.3%, CV3: 5.2%, and CV4: 3.2%. M = mercury and MC = mercury-cyanide.

The ANOVA revealed significant differences in wing shape among the different treatments and the control (*p* < 0.00), but not among the generations (*p* = 0.1). The control was different from all the treatments (*p* < 0.001) and the treatments with water affected by agriculture and mining mercury-cyanide had the highest morphological variations. Besides, the results from the assay with the sample from the reference site were different from the treatments with water affected by the anthropic activities (*p* < 0.001). The CVA explained 90.3% of the variance with the first two axes (Fig 7A). While between the positive end of CV2 and the negative end of CV1 the individuals treated with water affected by agriculture are grouped, the positive axis of CV2 and CV1 gather the individuals from the assay with water from the reference site and the control. Samples from both treatments with water samples from the two mining sites and the cattle raising site are grouped in the negative axis of CV2 and in the neutral point of CV1.

The first four CVAs show the most-relevant variations compared with the consensus of the shapes (Fig 7B–7E). While the light blue line shows the consensus of the shape of the Procrustes fit, the blue lines show the partial deformations represented in the four first canonic axes that explain 98.7% of the variance. The CV1 is the principal pattern of the wing’s shape variation (increased width and reduced basal part) with 68% of the total data variance (Fig 7B). This development instability pattern was registered in the groups treated with water from all the sites affected by anthropic activities, separating them from the control group and from the group treated with the reference sample. The CV2, with 22.3% of the total variation of the wing shape, was due to the increased width and reduced length in the basal part (Fig 7C). This development instability was evidenced in the treatments with samples of water from the sites affected by mining and cattle raising activities and from the reference site, separating them from the control group. The CV3, with 5.2% of the total wing-shape variation, is attributed to the increased length in the terminal part of the wing (Fig 7D) and was present in the assays with water affected by cattle raising and from the site without visible impact, separating them from the control. The CV4, with 3.2% of the total wing-shape variation, was due to the reduced width of the wing (Fig 7E) and was observed in the samples subjected to water from the WAI site and separating such from the control.

In summary, the morphology of the mentum and wing of *C*. *columbiensis* had greater variation in the treatment with mining mercury-cyanide, followed by agriculture, mining-mercury, cattle raising, and the WAI site, compared to the control.

### 3.3 Physical-chemical and hydrological variables and sampling sites

**Fig 8:**
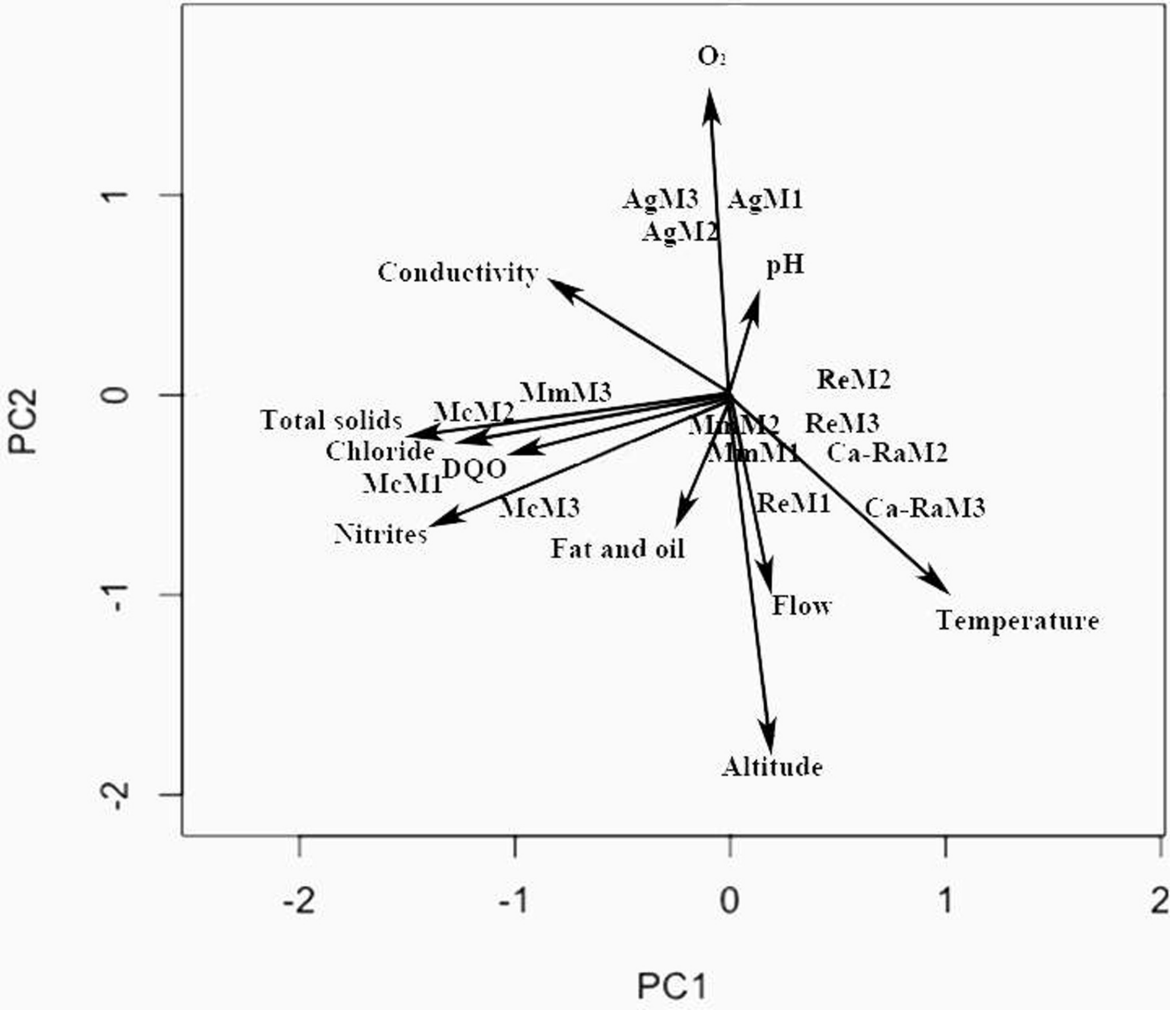
Principal components analysis of the physical-chemical and hydrological variables and the sites evaluated.

The principal components analysis managed to explain 54% of the total variance with the first two axes (Fig 8). Component I included 32.1% of the total data variation with two well-defined groups of variables. On one side, there are the sites with mining-mercury and mining-cyanide with total solids, nitrites, chloride, oxygen chemical demand, and conductivity. The other group has the sites with cattle raising and reference apparently related with temperature. Component II included 21.9% of the variation of all the data, also with two well-defined groups of variables. The agriculture site was related with the pH, oxygen concentration, and conductivity parameters, while the variables of altitude, flow, fats, and oils gather the site with mining mercury. In general, the principal components analysis suggests that pH, total solids, oxygen chemical demand, chloride, nitrite, sulfate, phosphate, and iron can be the variables being altered in the different sites with mining, cattle, and agriculture activities evaluated (Annex 1).

## 4 Discussion

The four types of deformities found in the study, like reduced number of teeth, absence of teeth, space between teeth, and fusion of teeth coincide with the findings by Arambourou *et al*., [2, 30] in *Chironomus riparius* from places subjected to industrial contamination and by Odume *et al*., [6] for *C*. *spp* in sites with agricultural and industrial contamination. The absence or increase of teeth has also been reported by other authors as one of the most-recurrent deformities in chironomidae exposed to organic compounds [2, 30, 27, 6]. Different studies have demonstrated that heavy metals and other toxic compounds can induce morphological defects in chironomid larvae [50, 51, 2, 30, 52]. This work found that chronic exposure of *C*. *columbiensis* to water samples from zones affected by mining, agricultural, and cattle raising activities also generated significant differences in the shape of the mentum and wing. These phenotypic changes are possibly associated to the presence of heavy metals and pesticides in aquatic ecosystems [6].

The space between teeth or Köhn’s gap is a deformity found in some *C*. *columbiensis* individuals from treatments of cattle raising and agriculture and, consequently, which show frequent use of pesticides. These results are coherent with those reported by Bisthoven *et al*., [53] & Groenendijk *et al*., [26]), who also found this type of deformity in organisms from sites with organic contamination. In addition, the third water sampling from the sites affected by agriculture, mining mercury and mining mercury and cyanide showed values of total coliforms and *E*. *coli* above the maximum permissible limits (Annex 1).

Lack of differences among the deformities of the mentum in the generations evaluated coincide with that found by Groenendijk *et al*., [26] in *C*. *riparius* exposed to sediments with heavy metals and Martínez *et al*., [54] with different species of Chironomidae from contaminated environments.

The results indicate that variations in shape of the *C*. *columbiensis* mentum can be used to evaluate the chronic effects of contamination on insect morphology [23, 55, 56]. In samples treated with water affected by mining mercury-cyanide, the highest variation was found in the shape of the mentum and wing in relation with the control. It has been demonstrated that the presence of heavy metals is one of the major triggers of the variation of the shape of chironomidae [53, 52, 51]. It is worth highlighting the importance of exploring the sub-lethal effects of contamination on natural populations. For this, it must be considered that some species can tolerate acute exposures to some compounds, but sub-lethal chronic exposures generate variations in their shape and in other biological traits [57].

Another treatment with greater variation in shape compared to the control was the agriculture treatment, which is an activity that affects the water resources, its fauna and flora associated [58, 59], given that the nutrient runoff [60] and use of pesticides [61, 62] filter into the water and are metabolized by the organisms, affecting the development of aquatic macroinvertebrates [63, 64]. Additionally, the site with agricultural activity showed a large amount of sediments present in this creek and increased pH compared with the other sites. Studies, like that by Mayer & Ellersieck [65] have demonstrated that pH has a greater modifying effect on the toxicity of contaminants to aquatic animals than any other factor.

According to the results, variation in the wing shape of *C*. *columbiensis* is a relevant variable to evaluate the effects of contamination because it showed changes in comparison with the control. The results from the present study differ from those by Hoffmann *et al*., [29] with *C*. *terpperi*, which did not find difference between the control group and the group exposed to pesticides. Furthermore, Arambourou *et al*., [30] found no significant differences in the wing of *C*. *riparius* among the control group and groups subjected to industrial contaminants. However, these results coincide with that reported by Campero *et al*., [66], who state that phenotypic defects must be detected, above all, in the adult-phase scenario of the samples due to increased energy expenditure of metamorphosis, a moment during which they are more susceptible to the exposure of toxic substances.

Given the low percentage (54%) of the variation explained with the first two PCA axes, no great importance was found of any physical-chemical parameter to reduce the dimensionality of the data matrix, hence, the PCA was used as an exploratory analysis to discover interrelations among said parameters. The groupings formed by both axes are coherent with that observed in the study, where the variables of chlorides, oxygen chemical demand, total solids, iron, nitrites, sulfates and phosphates had a direct relation. The highest values for these variables occurred in the mining zone.

During the samplings, the physical-chemical parameters of the water in the study zones were within the admissible quality criteria for the destination of the human and household resource (articles 38 and 39 of Decree 1594 of 1984). The only exceptions occurred in the third sampling, which for the agriculture zone evidenced values of total coliforms and *E*. *Coli* above those admissible (410,600 microorganisms/100 mL and 2,416.6 microorganisms/100 mL, respectively) and for the mining zone with values above those admissible for total coliforms (22,470 microorganisms/100 mL). This indicates that both zones are showing some type of temporary discharge that alters and creates fluctuations in the physical-chemical quality of the water.

## 5 Conclusions

Most studies on the use of water quality bioindicators indicate that environmental contamination generates effects on aquatic populations and communities. Here, we presented evidence that the phenotypic variations of the mentum and wing of *C*. *columbiensis* are a good morphological biomarker in the evaluation of the health of aquatic environments. Therefore, the use of biological assessment approaches, such as studies of morphological variations of the aquatic species based on geometric morphometry method, are valuables tools to complement the biomonitoring programs of water quality.

## Supporting information

S1 Fig Absence of teeth in the *Chironomus columbiensis* mentum under controlled conditions. The arrow indicates the region where the tooth is missing. A) Absence of the last lateral tooth of the right side. B) Absence of the last lateral teeth on the right side. C) Absence of the last lateral tooth on the left and right sides. D) Absence of teeth on the right, left, and medial sides.
(TIF)

S2 Fig Increased number of teeth of the *Chironomus columbiensis* mentum in controlled conditions. The arrow indicates the region with additional teeth. A) Medial region with four teeth. B) Medial region with five teeth. C) Medial region with four teeth. D) Medial region with six teeth.
(TIF)

S3 Fig Space between teeth of the *Chironomus columbiensis* mentum in controlled conditions. The arrow indicates the region with space between teeth. A) Space on the left side of the medial tooth. B) On the left side of the medial tooth with commissure of the medial tooth. C) On the right side of the medial tooth. D) Between the first and second right side lateral tooth.
(TIF)

S4 Fig Fusion of teeth of the *Chironomus columbiensis* mentum in controlled conditions. The arrow indicates where fusion of the teeth is taking place. A) Fusion of the left side internal lateral teeth. B) Fusion of the medial trifid tooth with inclination to the right. C) Fusion of the medial trifid tooth with absence of the two laterals of the medial trifid tooth. D) Fusions of teeth along the mentum.
(TIF)

S1 Table Preparation of the semi-soft reconstituted water to culture *Chironomus columbiensis* under controlled conditions. (DOCX)
