# Supplementary figures and images for: Incidence of deformities and variation in shape of mentum and wing of Chironomus columbiensis (Diptera, Chironomidae) as tools to assess aquatic contamination
Source: PLoS One. 2019 Jan 10;14(1):e0210348. doi: 10.1371/journal.pone.0210348 (PMC6328103; doi:10.1371/journal.pone.0210348)

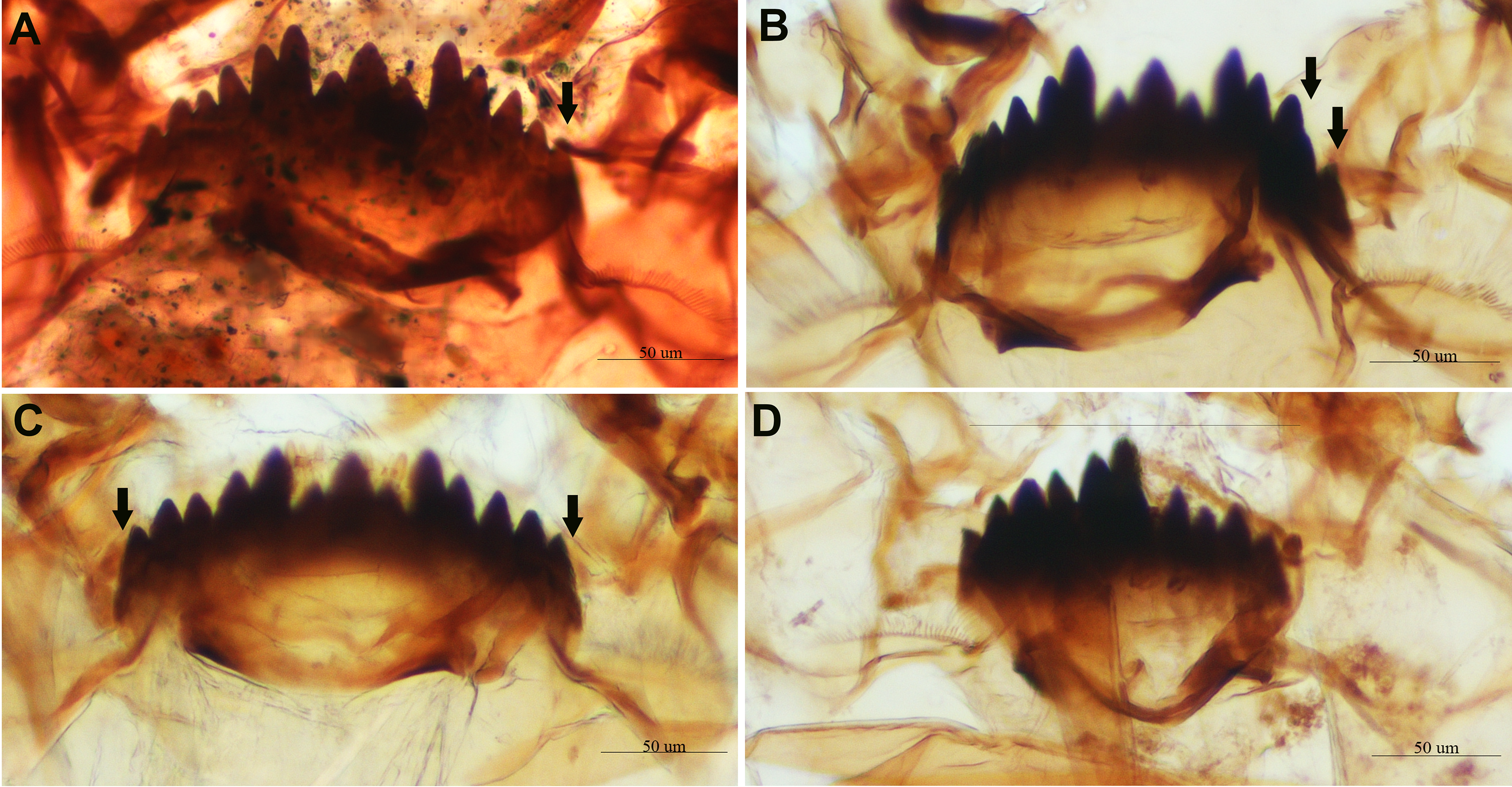

Supplement: S1 Fig — The arrow indicates the region where the tooth is missing. A) Absence of the last lateral tooth of the right side. B) Absence of the last lateral teeth on the right side. C) Absence of the last lateral tooth on the left and right sides. D) Absence of teeth on the right, left, and medial sides. (TIF) [file pone.0210348.s001.tif]

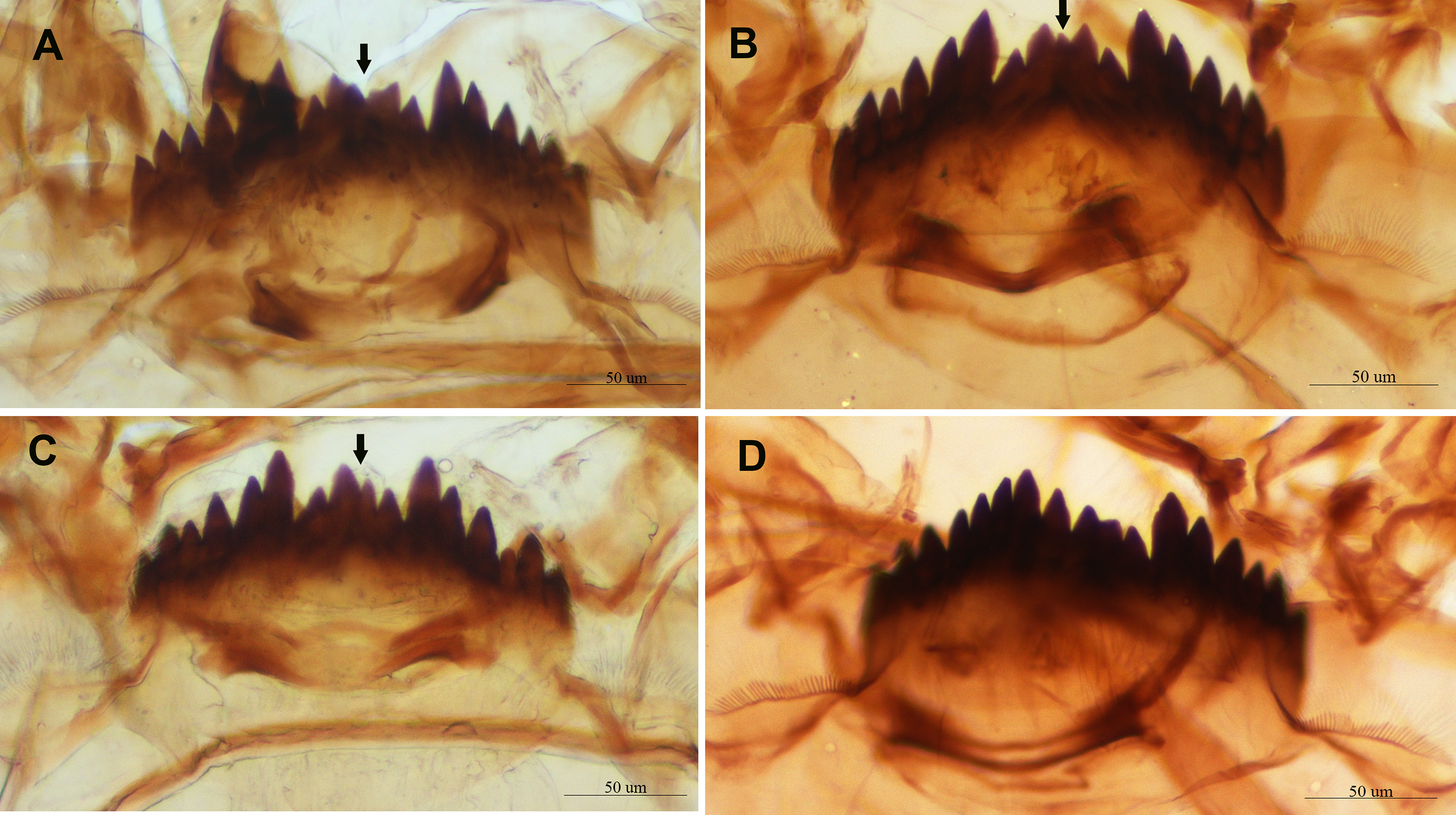

Supplement: S2 Fig — The arrow indicates the region with additional teeth. A) Medial region with four teeth. B) Medial region with five teeth. C) Medial region with four teeth. D) Medial region with six teeth. (TIF) [file pone.0210348.s002.tif]

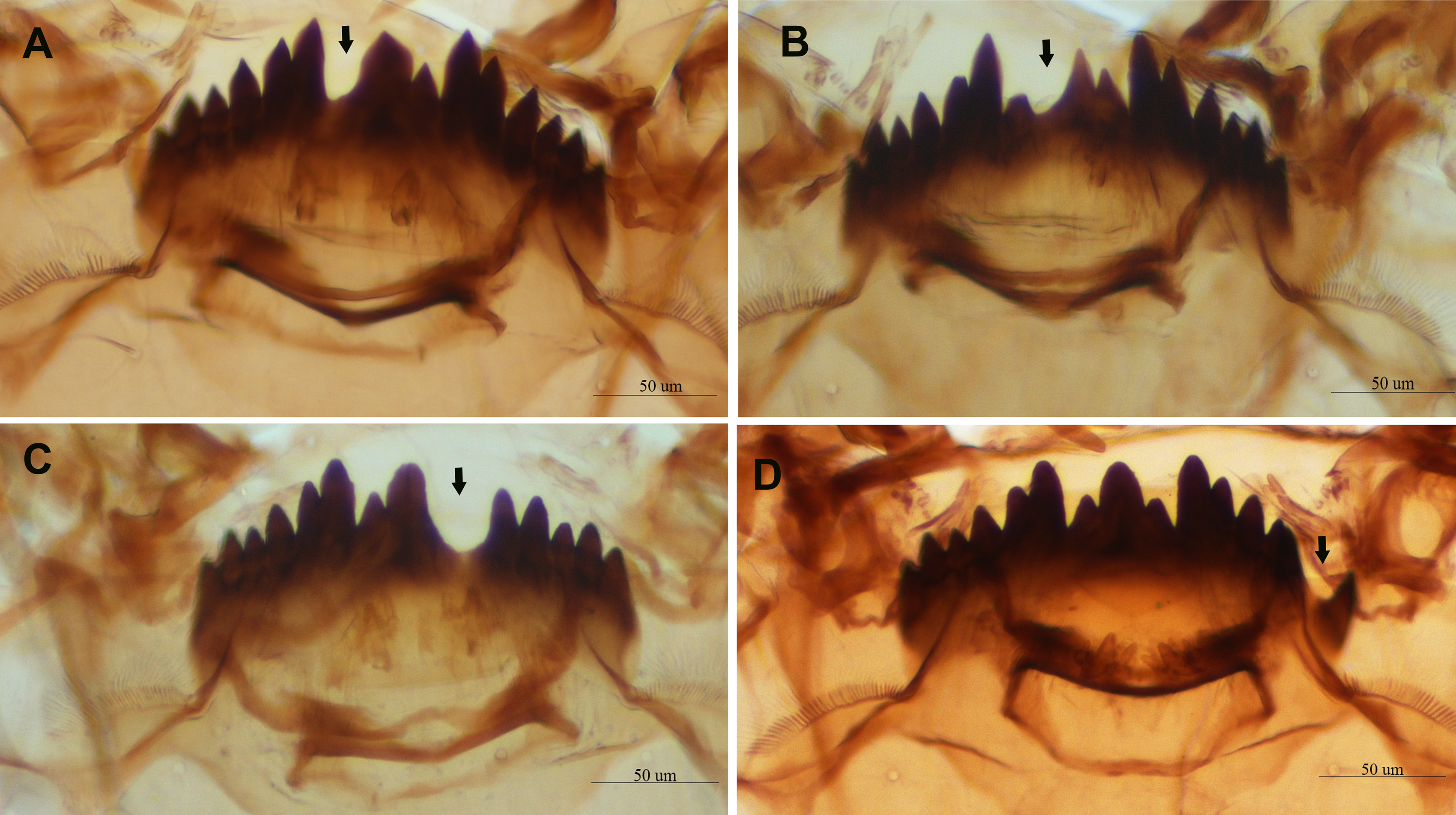

Supplement: S3 Fig — The arrow indicates the region with space between teeth. A) Space on the left side of the medial tooth. B) On the left side of the medial tooth with commissure of the medial tooth. C) On the right side of the medial tooth. D) Between the first and second right side lateral tooth. (TIF) [file pone.0210348.s003.tif]

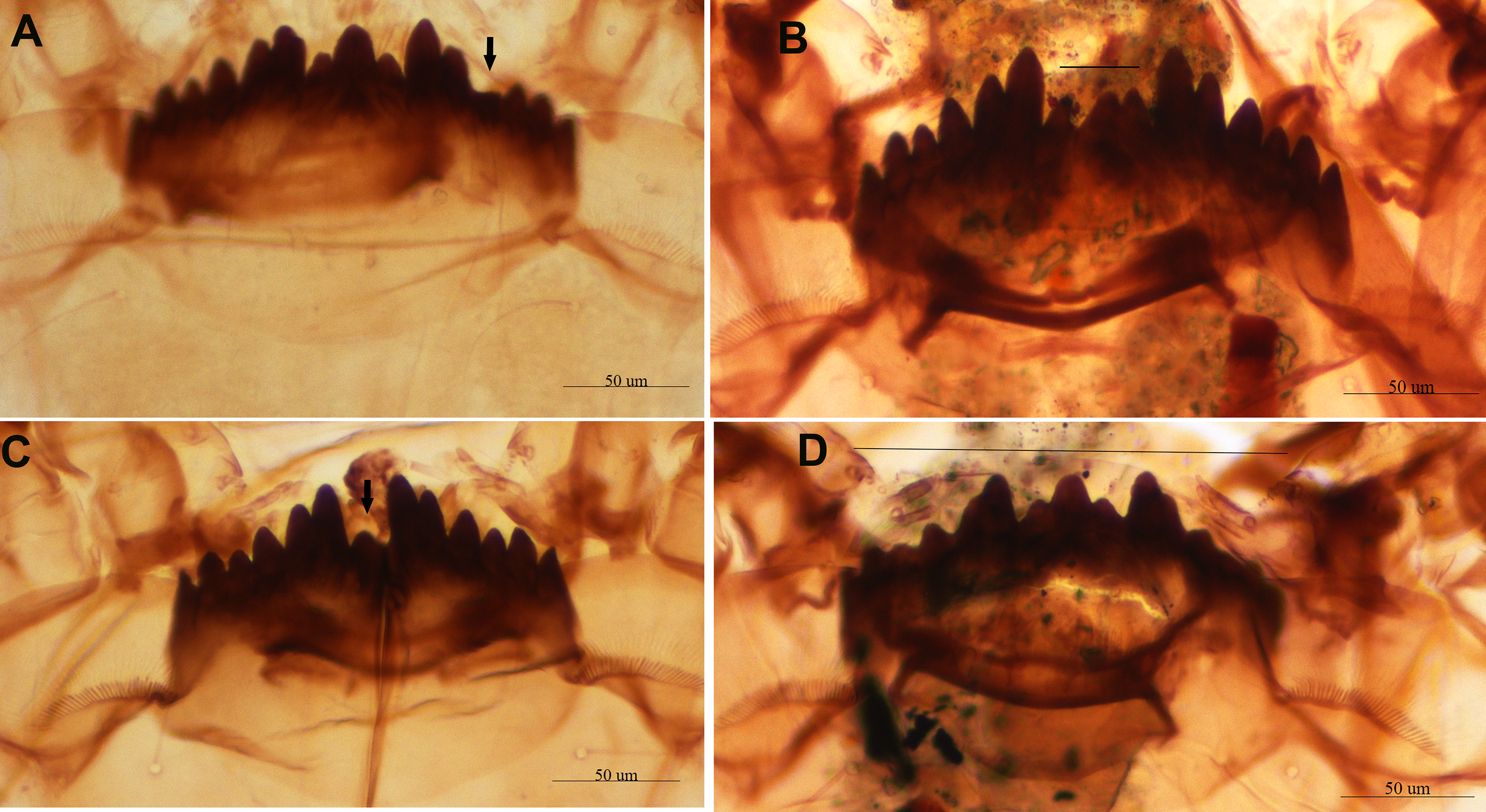

Supplement: S4 Fig — The arrow indicates where fusion of the teeth is taking place. A) Fusion of the left side internal lateral teeth. B) Fusion of the medial trifid tooth with inclination to the right. C) Fusion of the medial trifid tooth with absence of the two laterals of the medial trifid tooth. D) Fusions of teeth along the mentum. (TIF) [file pone.0210348.s004.tif]
