# Supplementary material for: Incidence of deformities and variation in shape of mentum and wing of Chironomus columbiensis (Diptera, Chironomidae) as tools to assess aquatic contamination
Source: PLoS One. 2019 Jan 10;14(1):e0210348. doi: 10.1371/journal.pone.0210348 (PMC6328103; doi:10.1371/journal.pone.0210348)
Supplement: S1 Table — (DOCX) [file pone.0210348.s005.docx]

| **Compounds** | | **SLN Stock** | **Preparation** |
| --- | --- | --- | --- |
| **Name** | **Nomenclature** | **Prepared in 500 ml** | **Amount per liter** |
| Potassium chloride | KCl | 3.6 g | 0.56 ml |
| Magnesium sulfate | MgSO_4_ | 13.5 g | 2.22 ml |
| Sodium bicarbonate | NaHCO_3_ | 43.2 g | 1.11 ml |
| Thiamin |  | 0.3 g | 0.25 ml |
| Hydrated calcium sulfate | CaSO_4_·2H_2_O |  | 0.06 g |

**S1 File. Preparation of the semi-soft reconstituted water to culture *Chironomus columbiensis* under controlled conditions.** **(DOCX)**
